# Exploring the causal relationships between rheumatoid arthritis and oral phenotypes: a genetic correlation and Mendelian randomization study

**DOI:** 10.3389/fgene.2024.1383696

**Published:** 2024-05-21

**Authors:** Jindan Shen, Yimei Lou, Liping Zhang

**Affiliations:** Department of Stomatology, Yaojiang Township Central Hospital, Zhuji, China

**Keywords:** rheumatoid arthritis, oral phenotypes, mouth ulcer, denture, Mendelian randomization, linkage disequilibrium score regression

## Abstract

**Background:**

Rheumatoid arthritis (RA) frequently presents with oral manifestations, including gingival inflammation, loose teeth, and mouth ulcers; however, the causal connections between these conditions remain unclear. This study aims to explore the genetic correlations and causal relationships between RA and prevalent oral phenotypes.

**Methods:**

Using summary data from genome-wide association studies of European populations, a cross-trait linkage disequilibrium score regression was conducted to estimate the genetic correlations between RA and six oral phenotypes. Subsequently, a two-sample Mendelian randomization (MR) approach was employed to assess the causal relationships, corroborated by various sensitivity analyses. Heterogeneity was addressed through the RadialMR method, while potential covariates were corrected using the multivariable MR approach.

**Results:**

A significant negative genetic correlation was detected between RA and denture usage (r_g_ = −0.192, *p* = 4.88 × 10^−8^). Meanwhile, a heterogenous causal relationship between RA and mouth ulcers was observed (OR = 1.027 [1.005–1.05], *p* = 0.016, *P*_heterogeneity_ = 4.69 × 10^−8^), which remained robust across sensitivity analyses. After excluding outlier variants, the results demonstrated robustly consistent (OR = 1.021 [1.008–1.035], *p* = 1.99 × 10^−3^, *P*_heterogeneity_ = 0.044). However, upon adjusting for covariates such as smoking, alcohol consumption, body mass index, and obesity, the significance diminished, revealing no evidence to support independent genetic associations.

**Conclusion:**

Genetically predicted RA increases the risk of mouth ulcers, and a negative genetic correlation is identified between RA and denture use. The observed heterogeneity suggests that shared immunological mechanisms and environmental factors may play significant roles. These findings highlight the importance of targeted dental management strategies for RA patients. Further clinical guidelines are required to improve oral health among vulnerable RA patients.

## 1 Introduction

Rheumatoid arthritis (RA) is a chronic autoimmune disorder predominantly affecting joints. Distinguished by inflammation and synovial proliferation, RA destroys cartilage and bone within joints, impairing physical functionality and decreasing the quality of life for those affected (Di Matteo et al., 2023). Globally, epidemiological evidence indicates an increasing incidence and considerable morbidity associated with RA, predominantly affecting older adults and observed more frequently in females (Almutairi et al., 2021). This demographic trend underscores the critical need for improved healthcare strategies to manage and mitigate the escalating burden of RA effectively (Heckert et al., 2024).

Oral manifestations of RA have attracted increasing attention owing to their prevalent occurrence (Silvestre-Rangil et al., 2016). Significantly, population-based research revealed a higher frequency of dental consultations in RA patients and an elevated prevalence of various dental pathologies (Juan et al., 2022). A meta-analysis demonstrated an increased risk of RA in individuals with periodontal disease, which was notably more pronounced in instances of prolonged disease duration (Qiao et al., 2020).

In biology, the phenotype is the observable characteristics of an individual, with a complex interplay of genetic makeup and environmental factors shaped by innate and acquired influences (Ren et al., 2023). Oral phenotypes, including gingival inflammation, loose teeth, and mouth ulcers, not only manifest specific oral diseases but also potentially indicate early systemic autoimmune disorders (Lee et al., 2021; Ye et al., 2024). While a growing body of evidence suggests a correlation between RA and oral phenotypes, the causality and underlying mechanisms of this association remain incompletely understood (Lopez-Oliva et al., 2024).

Within genetic epidemiology, exploring correlations between diseases and phenotypes from a genetic perspective is of paramount significance (Okada et al., 2014). Genetic correlation studies have profoundly transformed our understanding of complex diseases by identifying genetic variants contributing to disease susceptibility (Albiñana et al., 2023). Leveraging genetic variants as instrumental variables (IVs), Mendelian randomization (MR) analysis facilitates a systematic dissection of causal relationships within multifaceted etiological frameworks, which has contributed significantly to the pathology of RA (Chen et al., 2022). A recent MR study, for example, has rigorously identified a genetic association between seropositive RA and an increased risk of periodontal disease (Cheng et al., 2024). This technique proficiently navigates the complexities of confounding factors and reverse causation, presenting a methodologically rigorous and precise alternative to traditional epidemiological research paradigms (Gormley et al., 2023).

Here, we conducted a genetic correlation and MR study to dissect the causal relationships between RA and oral phenotypes. Our research provides deeper insights into the genetic underpinnings linking RA to oral health, guiding clinical practices and public health policies toward improved management of RA and its associated oral manifestations.

## 2 Materials and methods

### 2.1 Study design

**FIGURE 1:**
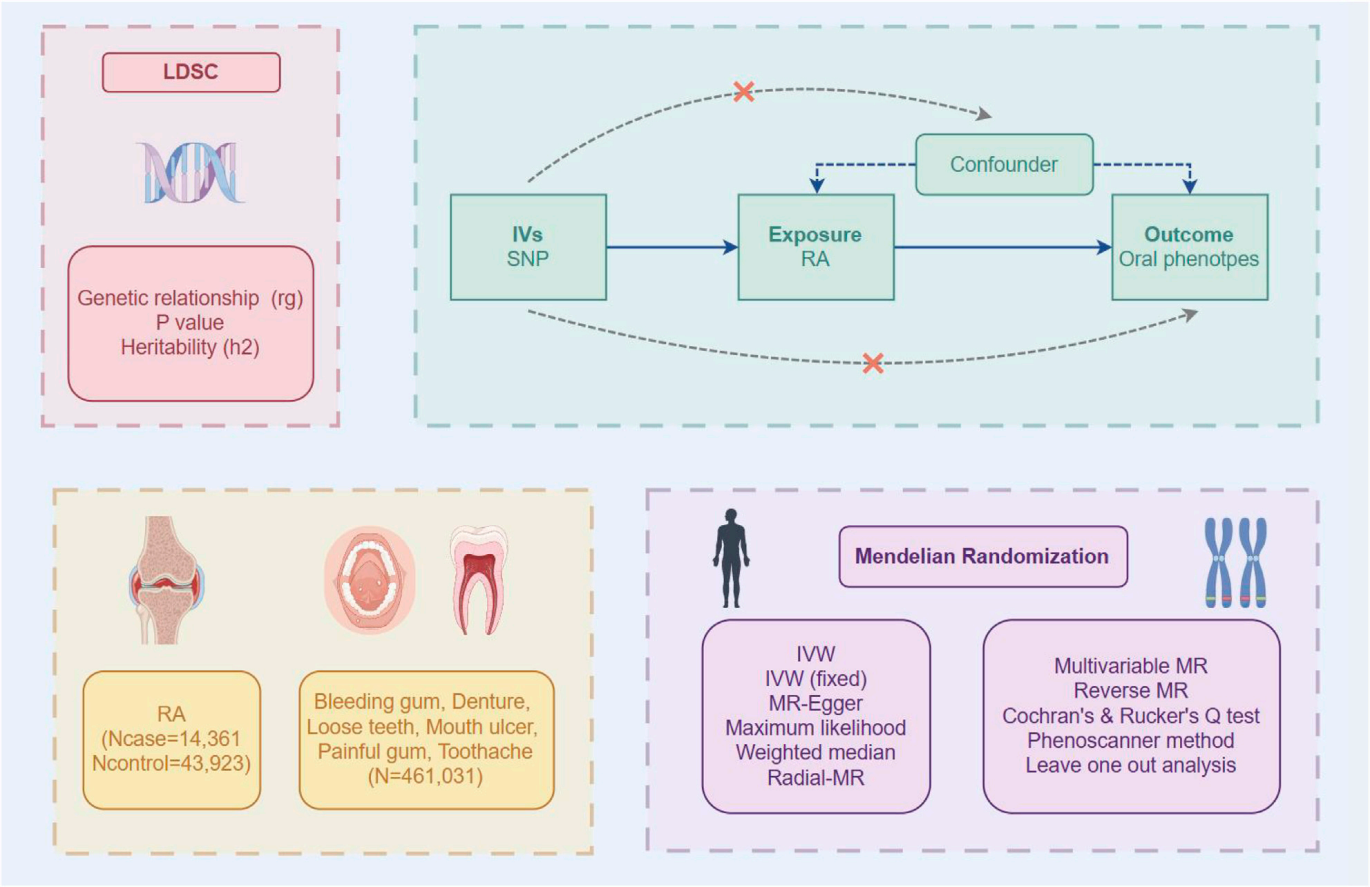
Overview of the design and methods used in this study. *LDSC* linkage disequilibrium score regression, *RA* rheumatoid arthritis, *SNP* single nucleotide polymorphism.

In this study, we first assessed the genetic correlations between RA and oral phenotypes through linkage disequilibrium score regression (LDSC). Then, we conducted a two-sample bidirectional MR analysis to investigate the potential causal relationships among these traits thoroughly. Additionally, we utilized the RadialMR tool to detect statistical outliers and elucidate the sources of heterogeneity. Ultimately, we employed multivariable MR (MVMR) to address potential biases arising from intermediate covariates (Figure 1).

This study adheres to the fundamental assumptions of MR research: 1) the IVs must be robustly associated with the exposure variable; 2) the IVs must not correlate with any confounding factors; and 3) the influence of the IVs on the outcome must be exclusively mediated through the exposure variable (Skrivankova et al., 2021). Since this study utilizes publicly accessible GWAS datasets, further ethical approval is not required. To correct for multiple comparisons, a Bonferroni-adjusted *p*-value threshold of 0.05/6 was employed. Statistical analyses were conducted utilizing the Mendelian randomization (version 0.9), TwoSampleMR (version 0.5.7), and RadialMR (version 1.1) packages within R software (version 4.2.3).

### 2.2 Data sources

Our study leveraged the largest GWAS summary data available and focused exclusively on European populations to minimize potential ethnic biases. The RA dataset originated from a GWAS meta-analysis of 18 European cohorts, comprising 14,361 RA patients and 43,923 controls, following the diagnostic criteria set by the American College of Rheumatology (Okada et al., 2014). Genome-wide summary data for oral phenotypes were obtained from 461,031 participants in the UK Biobank based on electronic questionnaires (http://www.nealelab.is/uk-biobank/) (Bycroft et al., 2018). This dataset detailed six oral phenotypes: denture use (77,714 cases), gum bleeding (60,210 cases), loose teeth (18,979 cases), toothaches (18,959 cases), gum pain (13,311 cases), and mouth ulcers (47,091 cases) (Shungin et al., 2019). Covariate GWAS datasets were sourced from the IEU OpenGWAS project (https://gwas.mrcieu.ac.uk/) using the following search queries: “Body mass index,” “Obesity,” “Cigarettes smoked per day,” and “Alcohol consumption” (Supplementary Table S1).

### 2.3 Selection of instrumental variances

We employed single nucleotide polymorphisms (SNPs) as IVs with a stringent selection criterion of *p* < 5 × 10^−8^. To mitigate potential biases induced by linkage disequilibrium (LD), SNPs were required to satisfy the criteria of an *r* ^2^ < 0.001 within a 10,000 kb LD distance. Additionally, we introduced the F-statistic to assess the strength of each SNP, calculated using the formula F = R^2^ × (N − 2)/(1 − R^2^), where R^2^ represents the proportion of variance explained by each IV, and N denotes the sample size. An F-value >10 indicates the absence of weak IV bias (Morales Berstein et al., 2022).

### 2.4 Linkage disequilibrium score regression

The LDSC tool (https://github.com/bulik/ldsc) was employed to investigate the heritability and genetic correlations between RA and oral phenotypes, comprising two main steps: 1) Heritability estimation (h^2^), which quantifies the proportion of trait variation due to genetic factors by analyzing SNP-based LD scores. 2) Genetic correlation calculation (r_g_), which assesses the extent of overlap in genetic determinants between RA and oral phenotypes (Bulik-Sullivan et al., 2015).

### 2.5 Two-sample Mendelian randomization

A two-sample MR was employed to estimate the genetic predictive influence of RA on oral phenotypes. The primary analysis used the inverse variance weighted (IVW) method. Heterogeneity was assessed using Cochrane’s Q and Rucker’s Q tests. A fixed effects model was prioritized when the *P*_Cochrane’s Q_ > 0.05; otherwise, a random effects model was adopted (Hemani et al., 2018). Three sensitivity analyses were performed to enhance the robustness of our findings (Burgess and Thompson, 2017; Xue et al., 2021): 1) Maximum likelihood estimation was utilized to assess the impact of genetic variants directly; 2) MR-Egger was applied to identify and correct for pleiotropy; 3) Weighted median approach provided a robust estimate amidst variability. Heterogeneity was managed by pinpointing outlier and influential SNPs through the RadialMR and leave-one-out plots (Bowden et al., 2018). Potential confounders were identified using the PhenoScanner database, and a reverse MR analysis was conducted to confirm the directionality (Kamat et al., 2019).

### 2.6 Multivariable Mendelian randomization

The MVMR analyses were conducted to evaluate the independent associations between RA and oral phenotypes, with the MVMR-IVW method as the primary approach. Three alternative MVMR-based sensitive analyses were employed, including MVMR-Egger, MVMR-median, and MVMR-LASSO, to account for pleiotropy and manage high-dimensional data (Grant and Burgess, 2021). Body mass index, obesity, smoking, and alcohol consumption were included as covariates due to their significant impacts on the immune system, potentially mediating the relationship between RA and oral phenotypes (Luo et al., 2023).

## 3 Results

### 3.1 Genetic correlations of RA on oral phenotypes

The liability-scale SNP heritability values (h^2^, h^2^se) were as follows: 2.21% (0.14%) for bleeding gum, 5.34% (0.21%) for denture, 1.27% (0.13%) for loose teeth, 2.95% (0.33%) for mouth ulcer, 0.64% (0.10%) for painful gum, and 0.68% (0.11%) for toothache. Significant genetic correlations were observed between RA susceptibility and dentures (r_g_ = −0.192, r_g_se = 0.035, *p* = 4.88 × 10^−8^) (Table 1).

**TABLE 1:** The heritabilities and genetic correlations between rheumatoid arthritis and oral phenotypes.

Exposure	h^2^	h^2^ se	Outcome	h^2^	h^2^ se	r_g_	r_g_ se	*P* val
RA	0.218	0.015	Bleeding gum	0.022	0.001	0.021	0.042	0.626
			Denture	0.053	0.002	−0.192	0.035	4.88 × 10^−8^
			Loose teeth	0.013	0.001	−0.060	0.056	0.283
			Mouth ulcer	0.030	0.003	0.002	0.041	0.951
			Painful gum	0.006	0.001	−0.018	0.065	0.783
			Toothache	0.007	0.001	0.018	0.068	0.791

*h* ^*2*^ heritability, *RA*, rheumatoid arthritis, *r*_*g*_ genetic correlation.

### 3.2 Causal relationships between RA and oral phenotypes

**FIGURE 2:**
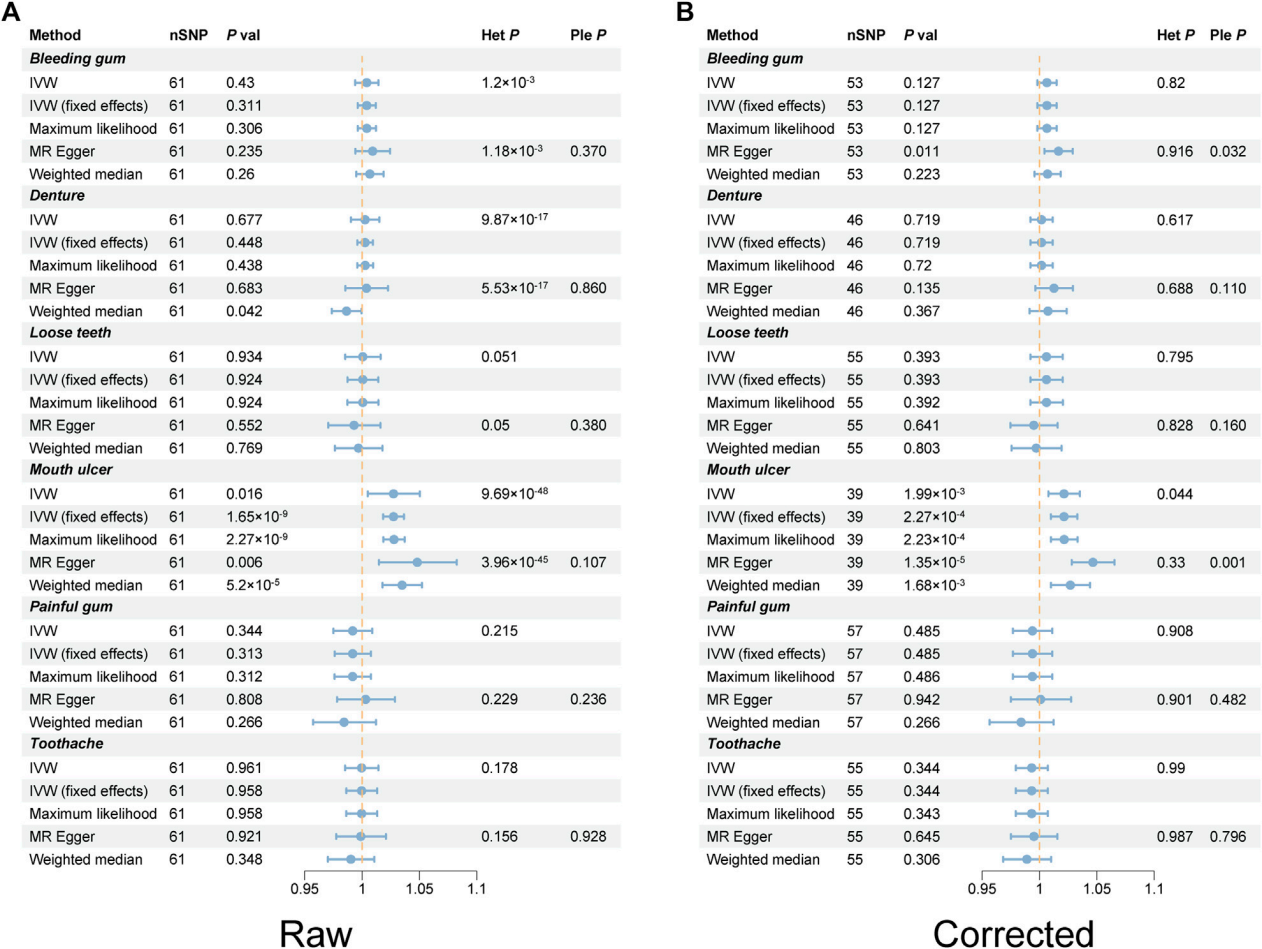
Forest plots of rheumatoid arthritis on oral phenotypes in TSMR. **(A)** Raw results. **(B)** Corrected result after removing outlier SNPs. *Het* heterogeneity, *IVW* inverse variance weighting, *Ple* pleiotropy, *RA* rheumatoid arthritis, *SNP* single nucleotide polymorphism, *TSMR* two-sample Mendelian randomization.

**FIGURE 3:**
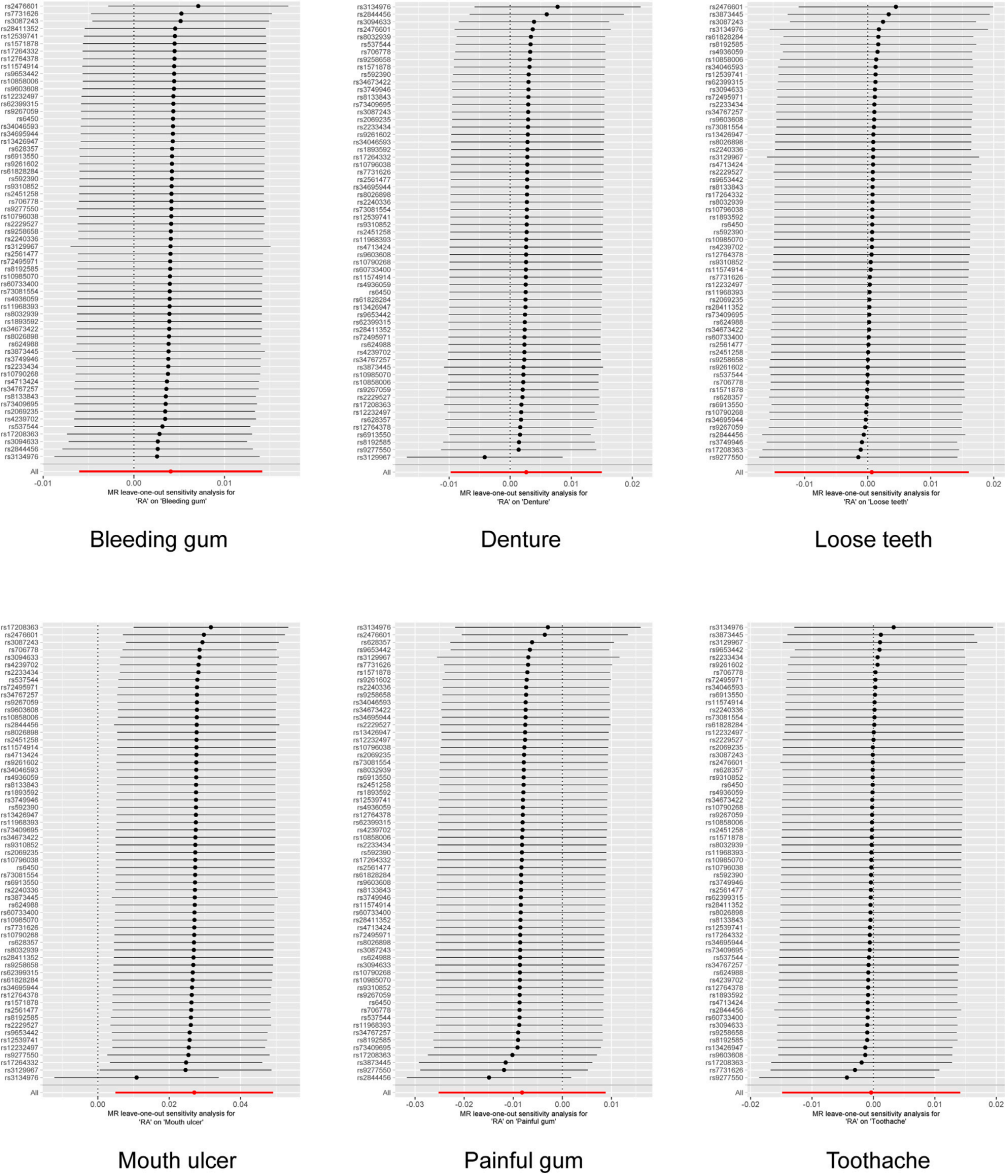
Leave one out analysis for the impact of individual SNPs on the association between rheumatoid arthritis and oral phenotypes.

Following rigorous screening, 61 SNPs were identified as IVs for RA, each possessing an F-value exceeding 10 (Supplementary Table S2). Most of these have been previously documented in the PhenoScanner database and are mainly associated with autoimmune diseases and blood routine indicators (Supplementary Table S3). Using the IVW method, a 2.74% increased risk of mouth ulcers in RA patients was revealed, according to the fixed model (OR [odds ratio] = 1.027, 95% CI [confidence interval] = 1.018–1.036, *p* = 1.65 × 10^−9^) and the random model (OR = 1.027, 95% CI = 1.005–1.05, *p* = 0.016). The MR-Egger method indicated a 4.81% increase in risk (OR = 1.048, 95% CI = 1.015–1.083, *p* = 0.006); the weighted median method demonstrated a 3.49% increase in risk (OR = 1.032, 95% CI = 1.018–1.052, *p* = 5.2 × 10^−5^); and the maximum likelihood method suggested a 2.78% increase in risk (OR = 1.027, 95% CI = 1.019–1.037, *p* = 2.27 × 10^−9^) (Figure 2A; Supplementary Table S4). The funnel plot demonstrated a symmetrical distribution of the selected SNPs (Supplementary Figure S1). The scatter plot analysis clearly showed the causality among SNPs (Supplementary Figure S2). Nonetheless, the leave-one-out plot revealed that several SNPs significantly affected the estimated causal relationship (Figure 3). The MR-Egger test detected no evidence of pleiotropy (Intercept = −0.007; *p* = 0.107). Furthermore, reverse MR analysis revealed no association between genetic susceptibility to mouth ulcers and an increased risk of developing RA (Supplementary Table S5).

### 3.3 Heterogeneity control and covariate correction

**FIGURE 4:**
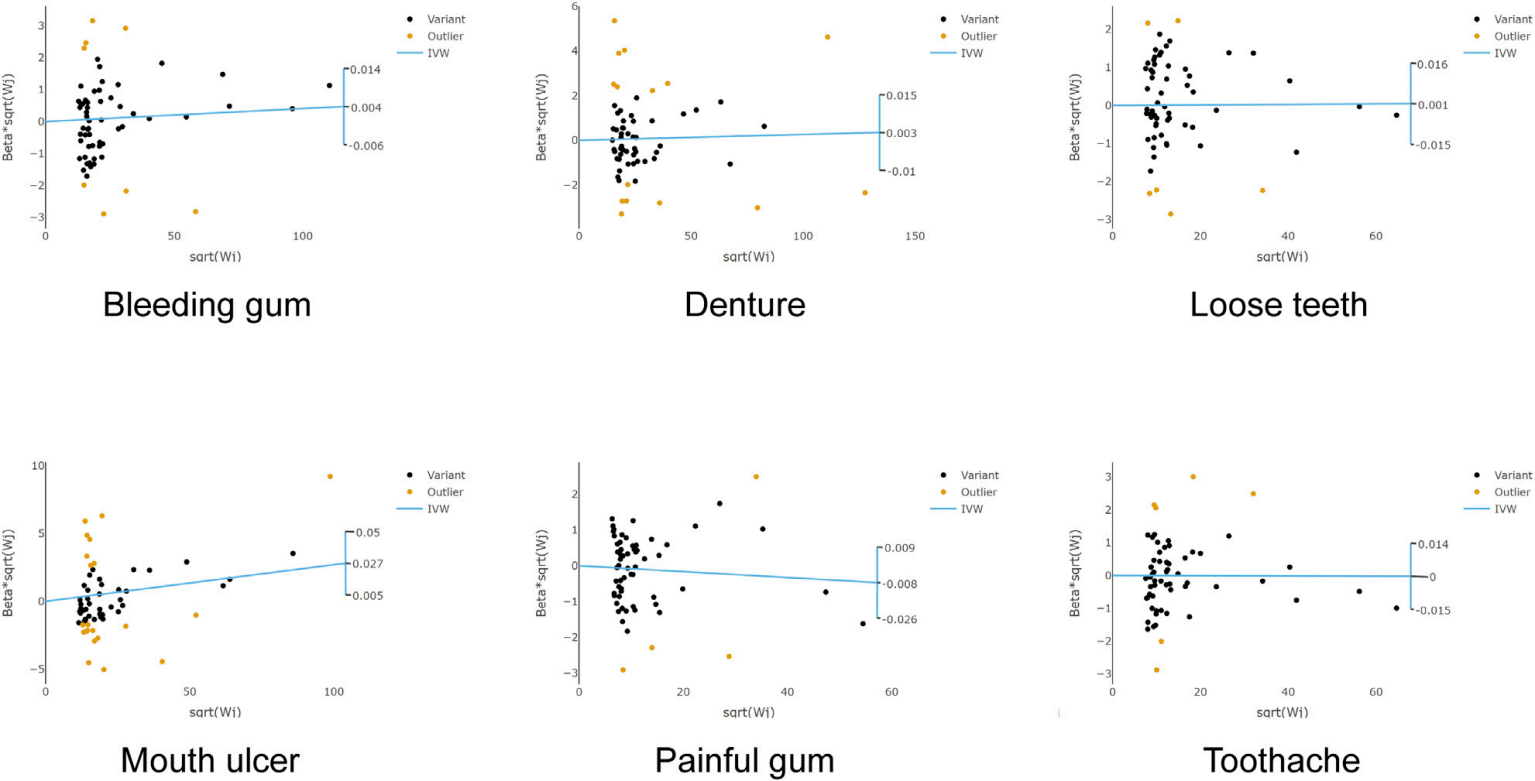
Scatter plots of the association between rheumatoid arthritis and oral phenotypes in RadialMR. Each genetic variant is represented by a point. *IVW* inverse variance weighting, *MR* Mendelian randomization.

**FIGURE 5:**
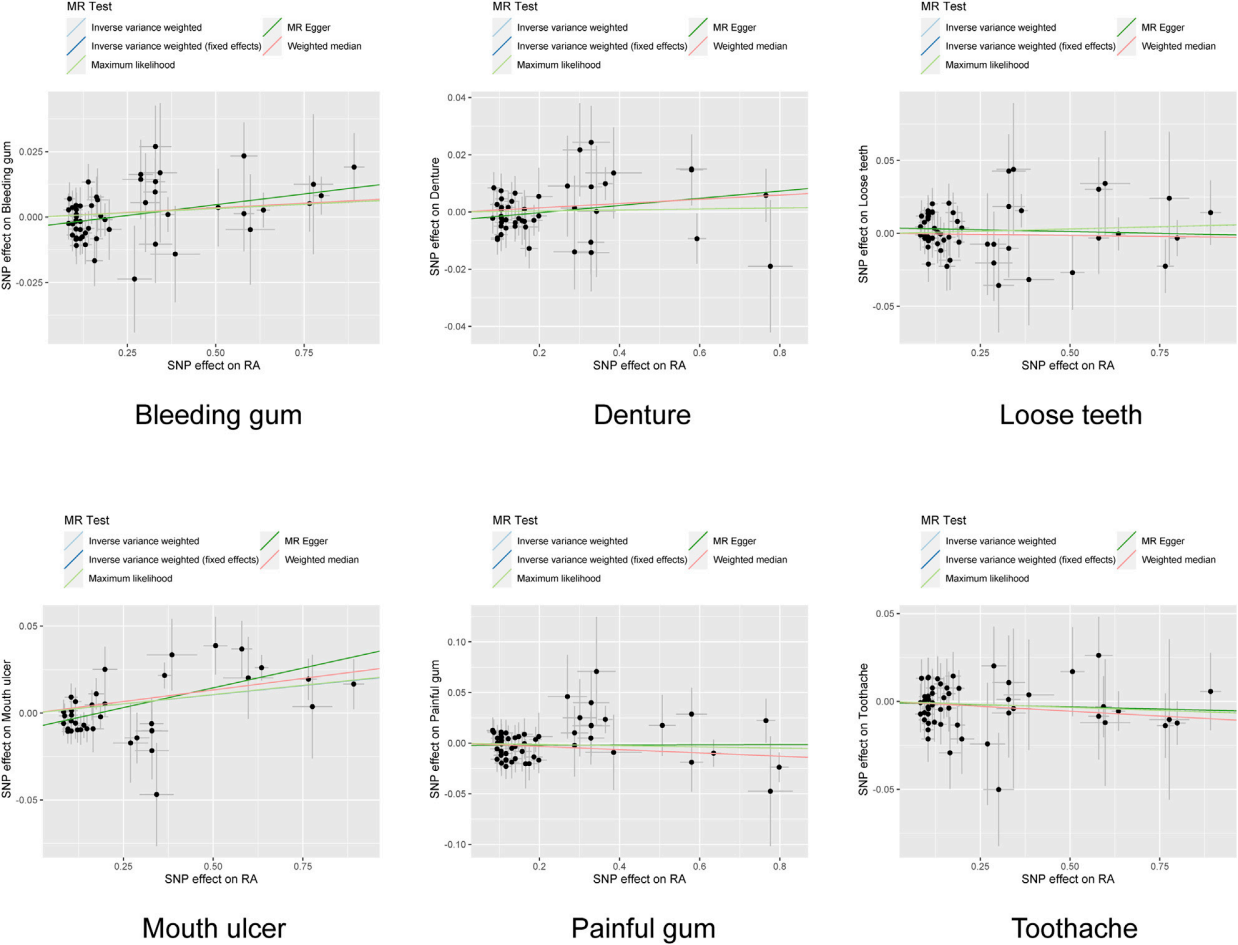
Scatter plots of the association between rheumatoid arthritis and oral phenotypes after the correction of outliers. Each black point represents an SNP, plotted by the estimate of SNP on RA (x-axis) and the estimate of SNP on oral phenotypes (y-axis). The slopes of each line represent the potential causal associations for each method. *IVW* inverse variance weighting, *RA* rheumatoid arthritis, *SNP* single nucleotide polymorphism.

To our interest, we noted a significant heterogeneity between RA and various oral phenotypes. Specifically, *P*_Cochran’Q_ = 0.001, *P*_Rucker’Q_ = 0.001 for bleeding gum, *P*_Cochran’Q_ = 9.87 × 10^−17^, *P*_Rucker’Q_ = 5.53 × 10^−17^ for denture, and *P*_Cochran’Q_ = 9.69 × 10^−48^, *P*_Rucker’Q_ = 3.96 × 10^−45^ for mouth ulcers. Following the utilization of the radialMR tool to remove outliers, heterogeneity significantly faded (*P*_Cochran’Q_ = 0.044, *P*_Rucker’Q_ = 0.33) (Figure 4). It was notable that the causal association between RA and the risk of mouth ulcer remained significant even after the corrections (Figure 5). Specifically, the IVW method indicated a 2.14% increase in risk with the fixed model (OR = 1.021, 95% CI = 1.001–1.033, *p* = 2.27 × 10^−4^), and the random model (OR = 1.021, 95% CI = 1.008–1.035, *p* = 1.99 × 10^−3^). The MR-Egger method revealed a 4.66% increase in risk (OR = 1.047, 95% CI = 1.028–1.065, *p* = 1.35 × 10^−5^); the weighted median method demonstrated a 2.69% increase in risk (OR = 1.027, 95% CI = 1.01–1.044, *p* = 1.35 × 10^−5^); and the maximum likelihood method suggested a 2.13% increase in risk (OR = 1.021, 95% CI = 1.01–1.033, *p* = 2.23 × 10^−4^) (Figure 2B; Supplementary Table S6). After adjusting for potential covariates via MVMR, the association between RA and mouth ulcers dissipated, alongside observing a significant degree of heterogeneity. Even though the MVMR-median results retained significance after adjusting for cigarettes (OR = 1.06, 95% CI = 1.023–1.1, *p* = 0.001) (Supplementary Table S7).

## 4 Discussion

In this study, we explored the genetic correlations and causal relationships between RA and six oral phenotypes. Our findings indicate a negative genetic correlation between RA and denture use, while a positive causal relationship exists between RA and mouth ulcers. These findings reveal complex interactions between RA and oral health, with significant heterogeneity highlighting the complexity of these associations.

Our study offers epidemiological evidence for the association between RA and mouth ulcers from a genetic perspective. Similarly, a cohort analysis indicated a higher incidence of mouth ulcers in the RA population [adjusted HR (hazard ratio) = 1.24, *p* = 0.003] (Juan et al., 2022). A real-world study revealed that mouth ulcers were associated with an increased risk of RA, suggesting that mouth ulcers may act as early indicators of systemic autoimmune conditions (HR = 1.19, *p* = 0.003) (Lee et al., 2021). Potential biological mechanisms could bridge the clinical associations observed between RA and mouth ulcers. Fundamentally, chronic immune dysfunction in RA patients, characterized by elevated activity of inflammatory cytokines, plays a critical role in developing mouth ulcers (Dc et al., 2021). Modulating or suppressing the abnormal immune response, either locally or systemically, could effectively manages various autoimmune or inflammatory oral conditions (Saccucci et al., 2018). Furthermore, medications frequently prescribed for RA, such as Methotrexate, may increase the risk of mouth ulcers as an adverse effect (Ramia De Cap and Michaels, 2021). Extended use of corticosteroids can also deteriorate oral mucosal health, thus elevating the risk of ulcer development (Best et al., 2018). However, Nawata et al. indicated that the uncontrolled nature of severe RA itself, rather than the side effects of medications, directly lead to mouth ulcers (Nawata et al., 2021). Additionally, RA can contribute to oral dryness, thereby compromising the mouth’s protective mucosal layer, which increases susceptibility to trauma and infection, potentially resulting in mouth ulcers (Aloyouny et al., 2022).

Our research also identified a significant negative genetic correlation between RA and denture use. This correlation can be attributed to RA patients’ oral structure and functionality alterations. Specifically, joint pain and impaired hand function in RA patients can significantly affect their ability to conduct standard oral hygiene practices, resulting in a marked decrease in the frequency of denture use (Kroese et al., 2022). Additionally, the oral mucosa of RA patients may be more susceptible to damage, increasing discomfort or pain when wearing dentures. This discomfort could further diminish their reliance on and usage of dentures (Andrade et al., 2018). Therefore, our study illuminates the interplay between RA and denture use, indicating that RA patients may encounter further challenges in oral health management. It also underscores the potential necessity of offering personalized oral healthcare services to this clinical practice (Maruoka et al., 2022).

Interestingly, we observed significant heterogeneity between RA and oral phenotypes. This heterogeneity is speculated to stem from the following aspects. On the one hand, RA and oral phenotypes may share specific immune signaling pathways, suggesting that similar immune mechanisms could trigger or exacerbate both conditions (Li et al., 2022). On the other hand, considering poor lifestyle habits, such as smoking, excessive alcohol consumption, and obesity, could further intensify the heterogeneity between them (Luo et al., 2023). We adopted a specialized approach within MR to tackle the potential effects of heterogeneity arising from various factors. Utilizing the MVMR analysis, we adjusted for covariates that could introduce heterogeneity to minimize their impact (Gormley et al., 2020). Additionally, to pinpoint potential outlier SNPs, we employed the RadialMR method. This method is recognized for its proficiency in diminishing heterogeneity in IVs, enhancing the accuracy of our findings (Hu et al., 2023).

Indeed, dentists are pivotal in the early identification and multidisciplinary management of RA. Oral symptoms are frequently observed and signify the initial clinical indicators of autoimmune diseases (Saccucci et al., 2018). Optimal management of RA requires multidisciplinary medical care, wherein dental practitioners may play an integral role in ensuring timely diagnosis and effective treatment (Mays et al., 2012). Specifically, our study confirmed the link between genetically predicted RA and mouth ulcers. These findings should deepen our comprehension of oral phenotypes associated with RA, contributing significantly to the early diagnosis, detection, prevention, and management of RA. This, in turn, is expected to improve the quality of life and health outcomes for individuals suffering from RA (Gomez-Casado et al., 2021).

However, several limitations require caution within the clinical translation. First, the UKB dataset depends on self-reported data, which may introduce biases and lack specificity when linking oral phenotypes to diseases. Second, the concentration on a European population limits the generalizability of our findings, given the variation in genetic, environmental, and cultural factors across different populations. Third, despite robust methodology, the heterogeneous nature of the observed relationship suggests intricate interactions or common mechanisms between RA and oral phenotypes rather than a direct cause-and-effect link.

## 5 Conclusion

Our study provides valuable insights into the associations between RA and specific oral phenotypes, indicating a negative genetic correlation between RA and denture use, as well as a positive causal relationship between RA and the risk of mouth ulcers. These findings furnish clues into the mechanisms linking RA to oral health, characterized by a complex interplay of genetic, lifestyle, and environmental factors. Nevertheless, the notable heterogeneity observed in these interactions highlights the necessity for future research to investigate the independent relationships.

## Data Availability

The original contributions presented in the study are included in the article/Supplementary Material, further inquiries can be directed to the corresponding author.
