# Supplementary material for: Exploring the causal relationships between rheumatoid arthritis and oral phenotypes: a genetic correlation and Mendelian randomization study
Source: Front Genet. 2024 May 21;15:1383696. doi: 10.3389/fgene.2024.1383696 (PMC11148354; doi:10.3389/fgene.2024.1383696)
Supplement: Supplementary file 2 [file Table1.DOCX]

Supplementary Figures

**Exploring the causal relationships between rheumatoid arthritis and oral phenotypes: a genetic correlation and Mendelian randomization study**

*Jindan Shen, Yimei Lou, Liping Zhang*

***Figure Legend***

**Supplementary Fig. 1** Funnel plots of the association between rheumatoid arthritis and oral phenotypes. Each genetic variant is represented by a point.

*IV* instrumental variable, *IVW* inverse variance weighting, *SE* standard error

**Supplementary Fig. 2** Scatter plots of the association between rheumatoid arthritis and oral phenotypes. Each black point represents an SNP, plotted by the estimate of SNP on RA (x-axis) and the estimate of SNP on oral phenotypes (y-axis). The slopes of each line represent the potential causal associations for each method.

*IVW* inverse variance weighting, *RA* rheumatoid arthritis, *SNP* single nucleotide polymorphism


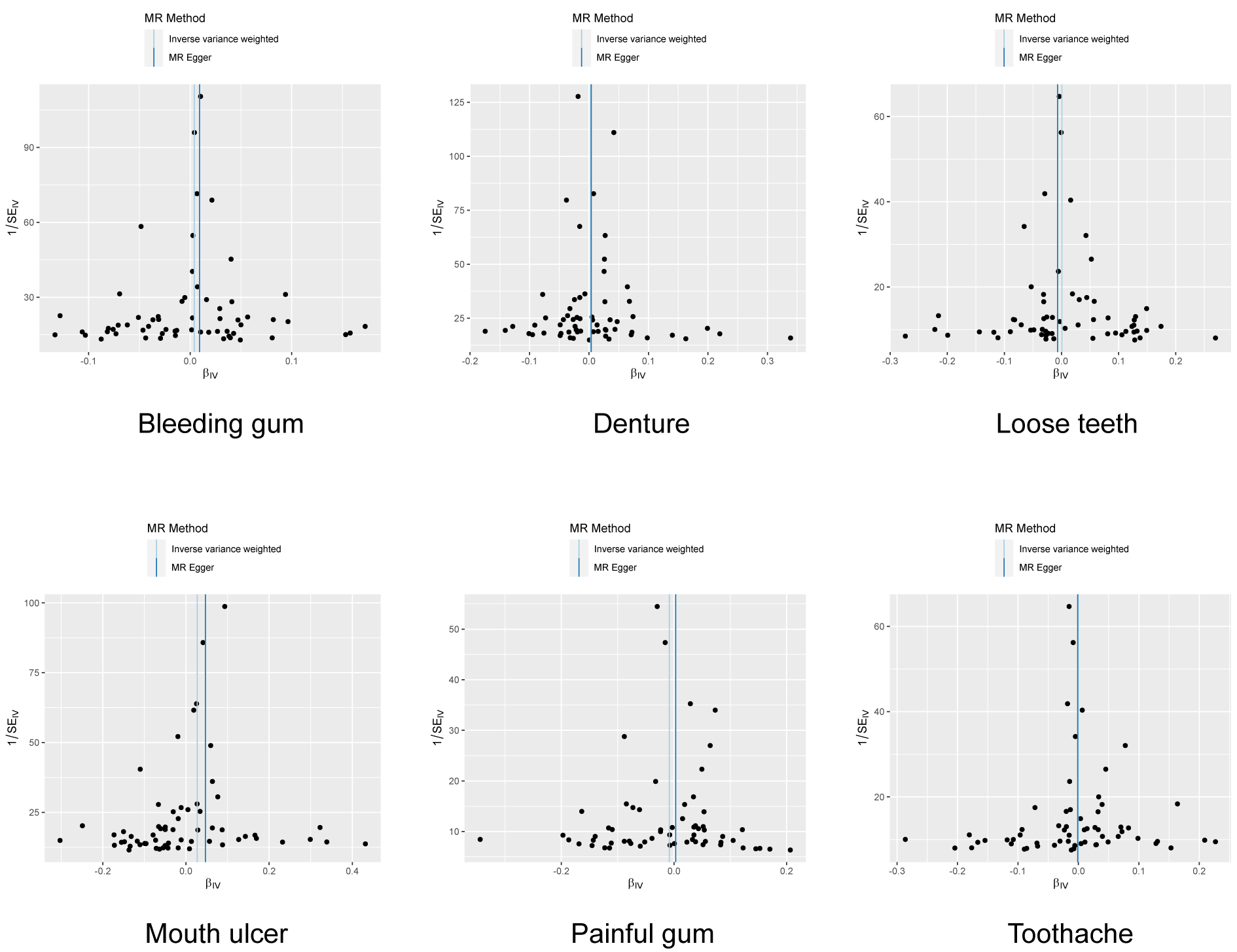


**SFig. 1**


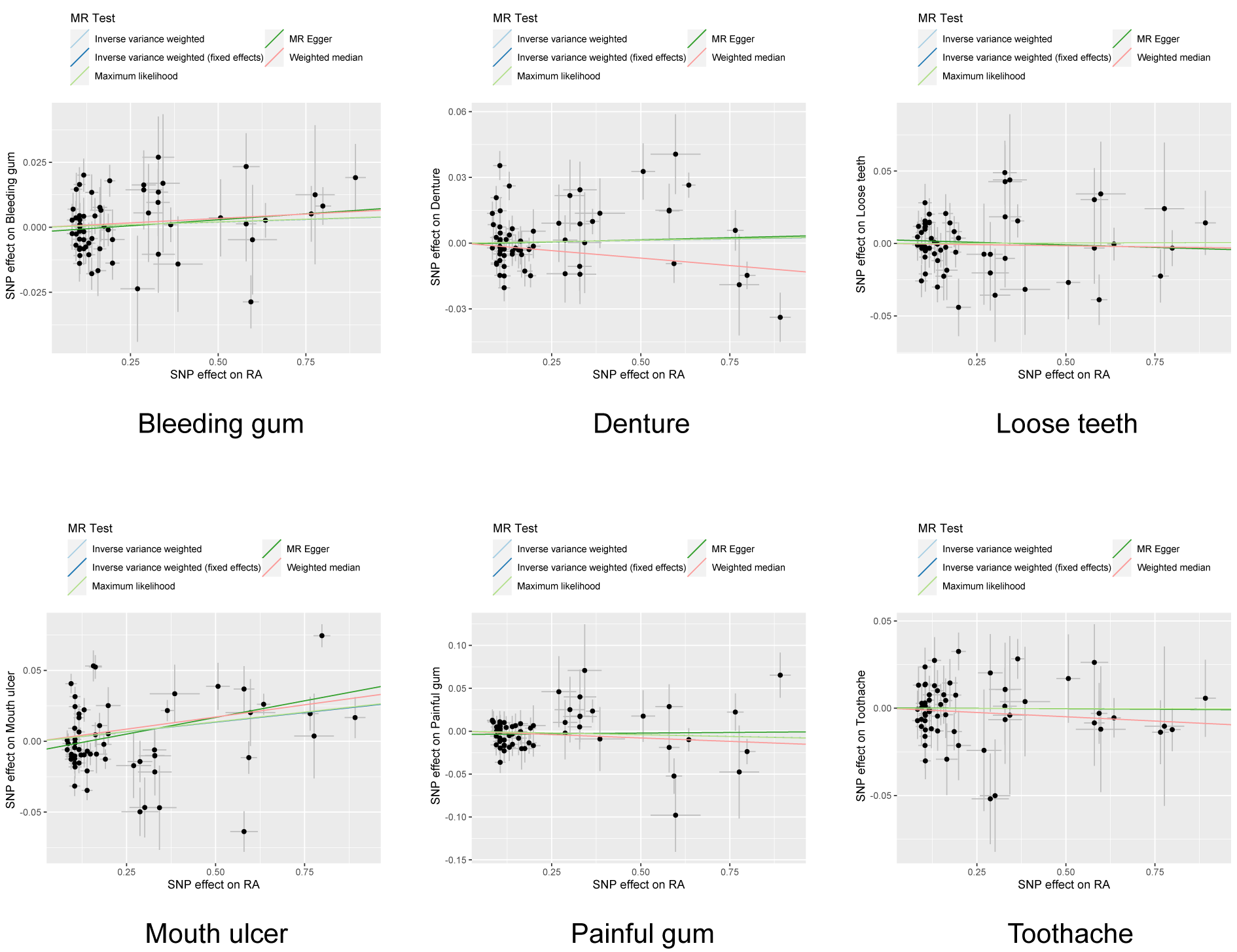


**SFig. 2**
